# Evaluation of the Antibacterial Activity and Cell Response for 3D-Printed Polycaprolactone/Nanohydroxyapatite Scaffold with Zinc Oxide Coating

**DOI:** 10.3390/polym12102193

**Published:** 2020-09-25

**Authors:** Yong Sang Cho, Hee-Kyeong Kim, Min-Soo Ghim, Myoung Wha Hong, Young Yul Kim, Young-Sam Cho

**Affiliations:** 1 Medical IT Convergence Research Section, Daegu-Gyeongbuk Research Center, Electronics and Telecommunications Research Institute (ETRI), 1, Techno Sunhwan-ro 10-gil, Dalseong-gun, Daegu 42994, Korea; yongsangcho@etri.re.kr; 2 Department of Mechanical Engineering, College of Engineering, Wonkwang University, 460 Iksandae-ro, Iksan, Jeonbuk 54538, Korea; peter_hee@naver.com (H.-K.K.); msghim5834@naver.com (M.-S.G.); 3 Department of Orthopedics, Daejeon St. Mary’s Hospital, The Catholic University of Korea, 64 Daeheung-ro, Jung-gu, Daejeon 34943, Korea; azirael99@naver.com; 4 Department of Mechanical Design Engineering, College of Engineering, Wonkwang University, 460 Iksandae-ro, Iksan, Jeonbuk 54538, Korea

**Keywords:** bone tissue engineering, antibacterial activity, 3D-printing, zinc oxide, kagome structure

## Abstract

Among 3D-printed composite scaffolds for bone tissue engineering, researchers have been attracted to the use of zinc ions to improve the scaffold’s anti-bacterial activity and prevent surgical site infection. In this study, we assumed that the concentration of zinc ions released from the scaffold will be correlated with the thickness of the zinc oxide coating on 3D-printed scaffolds. We investigated the adequate thickness of zinc oxide coating by comparing different scaffolds’ characteristics, antibacterial activity, and in vitro cell response. The scaffolds’ compressive modulus decreased as the zinc oxide coating thickness increased (10, 100 and 200 nm). However, the compressive modulus of scaffolds in this study were superior to those of other reported scaffolds because our scaffolds had a kagome structure and were made of composite material. In regard to the antibacterial activity and in vitro cell response, the in vitro cell proliferation on scaffolds with a zinc oxide coating was higher than that of the control scaffold. Moreover, the antibacterial activity of scaffolds with 100 or 200 nm-thick zinc oxide coating on *Escherichia coli* was superior to that of other scaffolds. Therefore, we concluded that the scaffold with a 100 nm-thick zinc oxide coating was the most appropriate scaffold to use as a bone-regenerating scaffold, given its mechanical property, its antibacterial activity, and its in vitro cell proliferation.

## 1. Introduction

The attachment of bacteria to various surfaces leads to the formation of biofilms, which are known to cause complications in humans [1,2]. During surgical operation, surgical site infections (SSIs) might arise as a result of the penetration of bacteria into the diseased area. In particular, SSIs following implant surgery are a major problem in the field of orthopedic surgery [3]. To preventing SSIs, studies have focused on implant devices with antibacterial functions using physical and chemical antibacterial methods. For the physical antibacterial method, nanopillars damage the cell wall of the bacteria when they attach to the surface, effectively killing the bacteria [4,5,6]. Hydrothermal synthesis and electrophoretic deposition were used to synthesize nanopillars on the surface of the specimen. However, this required high temperature, high pressure, and high electrical conduction properties [7,8,9,10]. Therefore, the physical antibacterial method was limited because of the biocompatible/biodegradable polymer’s lower melting point and subconductive characteristics. In contrast, the chemical antibacterial method uses metallic ions, which cause damage to the bacterial membrane and kills the bacteria [11]. This chemical antibacterial method could be applied to biocompatible/biodegradable polymeric scaffold via dip coating, electrophoretic deposition, metal-particle composite, or sputtering technique [12,13,14,15,16,17]. Accordingly, the chemical antibacterial methods could be used to fabricate a scaffold with antibacterial function with the use of metallic ions. Using silver, copper, or zinc as the metallic ions was attractive because of the relatively effective antibacterial activity each of them display [18]. In addition, zinc ions offer better cell adhesion and promote differentiation of mesenchymal stem cells along with the antibacterial activity [13]. Furthermore, the essential zinc ions in the bone tissue play a crucial role in the biochemistry of bone tissues [19]. Therefore, studies related to the antibacterial effect of zinc ions have been actively reported [20,21,22,23,24].

Moreover, in bone tissue engineering, scaffolds are very important because they can define the shape of the regenerated tissue and promote the proliferation and differentiation of cells by providing the various factors for cell response [25,26,27]. Moreover, the scaffold can provide mechanical support during bone regeneration in the bone-defect site [28,29]. Recently, studies have focused on fabricating bone-regenerating scaffolds with various functions with the use of 3D-printing technique. In our previous studies, a 3D-printed polycaprolactone/nanohydroxyapatite (PCL/nHA) scaffold with kagome structure was proposed to enhance the 3D-printed scaffold’s mechanical property [30,31]. In this study, we developed a 3D-printed kagome-composite (PCL/nHA) scaffold with an anti-bacterial zinc oxide coating with the use of a sputtering system to prevent surgical site infections. Herein, we assumed that the concentration of zinc ions released will be associated with the zinc oxide coating thickness on the surface of the scaffold. Therefore, to investigate the adequate thickness of the zinc-oxide coating, we evaluated the antibacterial effect and in vitro cell response of scaffolds with varying thicknesses of zinc oxide coating. We also compared the compressive modulus, water absorption, and concentration of zinc ions released from the scaffold with various thicknesses of zinc oxide coating.

## 2. Materials and Methods

### 2.1. Design of PCL/nHA Scaffold with a Periodic Kagome Structure

To fabricate a PCL/nHA scaffold with a kagome structure via a 3D-printing system, the periodic structure was designed using a computer-aided design program (CATIA V5, Dassault systems, Paris, France). The structural characteristics of scaffold with kagome structure are as follows: apparent dimension: 5 × 5 × 3.6 mm^3^, porosity: 50%, and apparent pore size: 500 µm. The pathway of 3D-printing system was created via an open-source program (Slic3r, V1.2.9, lead developer: Alessandro Ranellucci, Rome, Italy) from the STL file for the structure design of the abovementioned scaffold.

### 2.2. Preparation and Fabrication of PCL/nHA Scaffold with Kagome Structure and Zinc Oxide Coating

**Figure 1:**
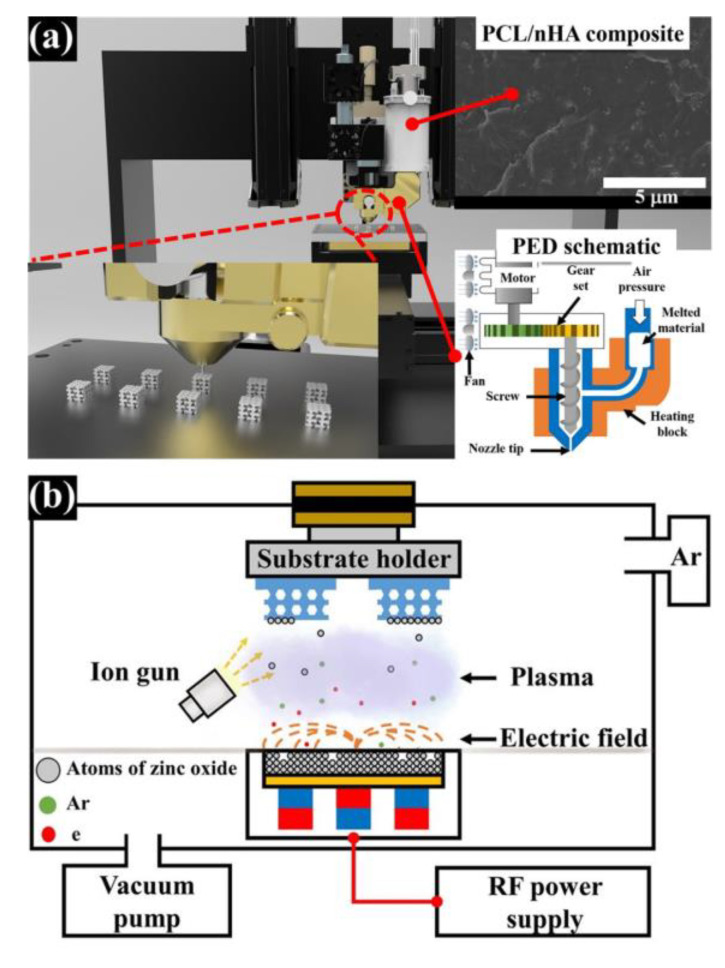
Schematics of the 3D-printed PCL/nHA scaffold with various thicknesses of zinc oxide coating: (**a**) 3D-printing system (material-extrusion system); (**b**) Radio frequency (RF) magnetron sputter system.

Polycaprolactone (PCL; M_w_ = 43,000–50,000 Da, Polysciences, Warrington, PA, USA) and nanosize hydroxyapatite (nHA; particle size: <200 nm, Sigma-Aldrich, St. Louis, MO, USA) were obtained to fabricate the composite material. To disperse nHA particles in the PCL matrix uniformly, a 5 *w*/*v* % PCL solution in dichloromethane (DCM; Daejung chemicals and materials, Siheung, Korea) was prepared by stirring for 1 h at 500 rpm. Then, 10 wt % (of total weight of composite material) nHA powders were added to the 5 *w*/*v* % PCL solution. The nHA particles in the PCL solution was dispersed using a sonicator (KFS-450N, Koprotec, Seoul, Korea) at 450 W for 45 min. Afterward, the dispersed PCL/nHA solution was transferred to each Teflon dish. The dispersed PCL/nHA solution in the dish was dried in a vacuum oven at 80 °C for 3 days. The prepared PCL/nHA bulk material was melted in a stainless-steel dispenser with a 100 µm nozzle at 88 °C for 1 h. The melted composite material was extruded by a single screw (PED system) at 45 rpm under the air pressure of 250 kPa and the PCL/nHA scaffold with kagome structure was simultaneously fabricated according to the created pathway, (Figure 1a). For the zinc oxide coating on the surface of the fabricated PCL/nHA scaffold, zinc oxide layers of varying thickness were deposited onto the fabricated scaffold using an RF (radio frequency) magnetron sputter system (system made by the photonic energy device laboratory at Korea Polytechnic University, Siheung, Korea) with a zinc oxide target source (high purity chemicals, Sakado, Japan) at 70 W in argon gas atmosphere for 56 s (layer thickness of 10 nm), 562 s (layer thickness of 100 nm), or 1125 s (layer thickness of 200 nm). The parameters of the fabricated PCL/nHA-ZnO scaffolds are summarized in Table 1. Furthermore, the PCL/nHA scaffold, PCL/nHA scaffold with 10 nm zinc oxide coating, PCL/nHA scaffold with 100 nm zinc oxide coating, and PCL/nHA scaffold with 200 nm zinc oxide coating are referred to as “PCL/nHA scaffold”, “PCL/nHA-ZnO10 scaffold”, “PCL/nHA-ZnO100 scaffold”, and “PCL/nHA-ZnO200 scaffold”, respectively.

### 2.3. Analysis of the Characteristics of Fabricated Scaffolds

The morphological characteristics of the fabricated scaffolds were investigated via a field emission scanning electron microscope (FE-SEM; S-4800, Hitachi, Tokyo, Japan). For cross-sectional morphology observation, the composite scaffolds were immersed in liquid nitrogen at –196 °C for 1 min. Afterward, the frozen composite scaffold was broken. The surface of the fabricated scaffolds and the cross section of broken composite scaffolds were coated with platinum for 120 s. The scaffolds were fixed on a plate and the morphology of the scaffolds were investigated in a vacuum chamber. For cross-sectional morphology, the composite and zinc oxide layers were colorized using an illustration software program (Photoshop CS6 version 13.0, Adobe systems, San Jose, CA, USA). Additionally, the chemical components of the fabricated scaffolds were analyzed via an energy dispersive spectroscopy (EDS; EX-250, Horiba, Tokyo, Japan) at 10 kV (two specimens were used for each scaffold type).

Compressive modulus of each scaffold was measured via the universal testing machine (UTM; Model E42, MTS, Eden Prairie, MN, USA) at a constant strain rate of 1 mm/min with 5 kN loading cell. The compressive modulus was calculated within 1% strain of the S-S curve (six specimens were used for each scaffold type). Additionally, to analyze the change in molecular weight of PCL in the fabricated scaffold with various thicknesses of zinc oxide layer, the weight-average molecular weight was investigated via gel permeation chromatography (GPC; ACQUITY APC, Waters, Milford, MA, USA) (two samples were used for each scaffold type). The change in chemical composition of the fabricated scaffolds by zinc oxide coating process were analyzed using Fourier transform infrared spectroscopy (FT-IR; 6300FV, JASCO, Tokyo, Japan) (two samples were used for each scaffold type).

### 2.4. Measuring Water Absorption Ability and the Concentration of Zinc Ions Released from the Scaffolds

The water absorption ability of the scaffolds was measured as follows: the scaffolds were immersed in bottles filled with 6 mL of pH 7.4 phosphate-buffered saline (PBS; Gibco, Gaithersburg, MD, USA) after the scaffolds initial masses were measured. Subsequently, bottles were placed in an incubator at 37 °C for 12 h. Thereafter, the soaked scaffolds were taken out and the masses were measured. The water absorption percentages of the scaffolds were calculated using Equation (1). For each scaffold type, five scaffolds were used.

(1) $$\mathrm{Water}\;\mathrm{absorption}\;\left(\%\right)\;=\;\frac{W_{12h}-W_{0}}{W_{0}}\times 100,$$

where *W*_12h_ is the weight of soaking scaffold after immersion in PBS solution for 12 h, and *W*_0_ is the initial weight of fabricated scaffold.

To measure the concentration of zinc ions released from the fabricated scaffolds with various thicknesses of zinc oxide coating, the fabricated scaffolds were immersed in the 1 mL of deionized water. Afterward, the deionized water was maintained in an incubator at 37 °C for 1, 3, or 7 days. The concentrations of zinc ions released into the deionized water were investigated via inductively coupled plasma optical emission spectrometer (ICP-OES; 700-ES, Varian, Palo Alto, CA, USA) (three specimens were used for each scaffold type).

### 2.5. Assessment of In Vitro Antibacterial Activity of PCL/nHA-Zinc Oxide Scaffold

To evaluate the antibacterial activity of the PCL/nHA-ZnO scaffold, Gram-negative *Escherichia coli* (*E. coli*, KTCT 2441) bacteria was purchased from Korean collection for type cultures (KTCT, Jeongeup, Korea). *E. coli* is one of the bacteria that cause surgical site infections in the orthopedic surgery field [32,33]. Before the anti-bacterial test, each bacteria suspension was cultured in 5 mL liquid nutrient medium (LB broth, LabM Ltd., Heywood, UK) for 18 h at 170 rpm on an orbital shaker (SHO-1D, daihan scientific co., Wonju, Korea) in the incubator (JSGI-50T, JS Research Inc., Gongju, Korea) at 37 °C. Bacteria growth of the cultures was analyzed via optical density measurements at 600 nm (OD600) with a spectrophotometer (AS ONE, Osaka, Japan) until the OD600 = 0.1. The scaffolds were sterilized using 70% EtOH overnight under UV light and were washed three times with a phosphate-buffered saline (PBS, Sigma-Aldrich, Gaithersburg, MD, USA). The sterilized scaffolds were fixed in the 96 well-plate. The adjusted 700 µL *E. coli* suspension (OD600 = 0.1) was seeded onto the scaffolds. Then, the bacteria was grown for 24 h at 170 rpm on an orbital shaker in the incubator at 37 °C. A colony-forming unit (CFU) assay was performed to quantify the antibacterial rate of the scaffold. The cultured scaffold was transferred to a conical tube filled with 1 mL of PBS. Afterward, the conical tube was vortexed for 10 min to separate the attached bacteria from the scaffold. This bacteria suspension was diluted serially and spread onto LB agar plates and cultured for 18 h. The number of viable bacterial colonies was counted (three specimens were used for each scaffold type). To evaluate the anti-bacterial ability, the antibacterial rate was calculated via the Equation (2).

(2) $$\mathrm{Anti}-\mathrm{bacterial}\;\mathrm{rate}\;\left(\%\right)\;=\;\frac{{\mathrm{CFU}}_{\mathrm{control}}-{\mathrm{CFU}}_{\mathrm{solution}}}{{\mathrm{CFU}}_{\mathrm{control}}}\times 100,$$

where *CFU_control_* is the average number of bacteria in the PCL/nHA scaffold; and *CFU_solution_* is the number of bacteria in the PCL/nHA-ZnO10 scaffold, PCL/nHA-ZnO100 scaffold, or PCL/nHA-ZnO200 scaffold.

The morphologies of live and dead bacteria were evaluated on the surface of scaffolds cultured for 24 h using a field emission scanning electron microscope (FE-SEM; S-4800, Hitachi, Tokyo, Japan). The cultured scaffold was carefully washed 2 times via PBS to wash away the nonadherent bacteria. The bacteria attached to the scaffold was fixed using paraformaldehyde (Daejung chemicals and materials, Siheung, Korea) for 15 min at room temperature and then observed. In addition, bacteria viability on the surface of the scaffold according to the releasing zinc ion was assessed via a live/dead bacterial viability kit (L7012, Thermo Fisher Scientific, Waltham, MA, USA). The stained suspensions were incubated for 15 min in the dark at room temperature. Subsequently, 50 µL of the stained suspension was spread on a slide glass. The slide glass was observed via fluorescence microscope (Axio scope A1, Carl Zeiss, Gottingen, Germany).

### 2.6. Assessment of In Vitro Cell Viability and Growth on PCL/nHA-Zinc Oxide Scaffold

After a few passages, cultured saos-2 cells (human osteosarcoma cell line, ATCC, Manassas, VA, USA) were trypsinized with trypsin-EDTA (0.05 *w*/*v*% trypsin and 0.02 *w*/*v*% ethylenediaminetetraacetic acid) (Gibco BRL, Grand Island, NY, USA). The scaffolds were sterilized using 70% EtOH overnight under UV light and were washed three times using a phosphate-buffered saline (PBS; HyClone, USA). Cell suspensions of saos-2 (cells/scaffold 1 × 10^5^) were seeded onto the surface of the prewetted scaffolds. The scaffolds were left undisturbed in an incubator for 30 min to enable the attachment of cells to the scaffold. After 30 min, the saos-2 media was added, and the cells were maintained in Dulbecco’s modified Eagle’s medium (DMEM; HyClone, Logan, UT, USA) supplemented with 10% of fetal bovine serum (FBS; HyClone, Logan, UT, USA), 100 U/mL of penicillin (Gibco BRL, Grand Island, NY, USA), and 100 µg/mL of streptomycin (Gibco BRL, Grand Island, NY, USA). The saos-2 cell cultures were maintained at 37 °C with 95% air and 5% CO_2_. The medium was changed every 3 days. Analytical assays were performed on days 1, 7, and 14. To investigate the seeding efficiency and cell growth in the scaffolds, viable cells were measured using the cell counting kit-8 assay kits (CCK-8; Dojindo, Kumamoto, Japan) following the manufacturer’s instructions. The absorbance was measured at 450 nm using a microplate reader every 30 min. The cell proliferation data are presented as the mean optical density values from three different specimens.

To assess cell proliferation on the different scaffolds, DNA content of saos-2 cells cultured for 1, 3, or 7 days was measured. The cell cultured scaffolds were washed with PBS 3 times, digested overnight at 56 °C in proteinase K solution (Thermo Fisher Scientific, Waltham, MA, USA) in Tris-EDTA pH 7.5 (Biosesang, Seongnam, Korea), and then stored at −80 °C. The next day, the frozen scaffolds were thawed at room temperature and sonicated for 50 s to release cellular DNA into the solution. After centrifugation at 13,000 rpm for 10 min at 4 °C, the supernatant was transferred to a new tube. DNA content was measured in triplicate using a spectrophotometer (Nanodrop ND 1000, Thermo Fisher Scientific, Waltham, MA, USA).

The viability of the saos-2 cells on the scaffolds was evaluated by utilizing a Live/Dead viability kit (Molecular Probes, L-3224, Eugene, OR, USA). Calcein AM dye, which fluoresces green, was reacted with the live cells, while ethidium homodimer-1 dye, which fluoresces red, was reacted with the dead cells. After 3 or 7 days of culture, the scaffolds were observed using a fluorescence microscope (Nikon, Tokyo, Japan).

### 2.7. Statistical Analysis

The obtained data are represented as the mean ± standard deviation. Data were analyzed by the single factor analyses of variance (ANOVA) using a statistical analysis software (SPSS version 21.0, Armonk, NY, USA). Data were considered statistically significant when *p* < 0.05.

## 3. Results

### 3.1. Investigation of the Scaffold’s Characteristics According to the Zinc Oxide Coating Thickness 

**Figure 2:**
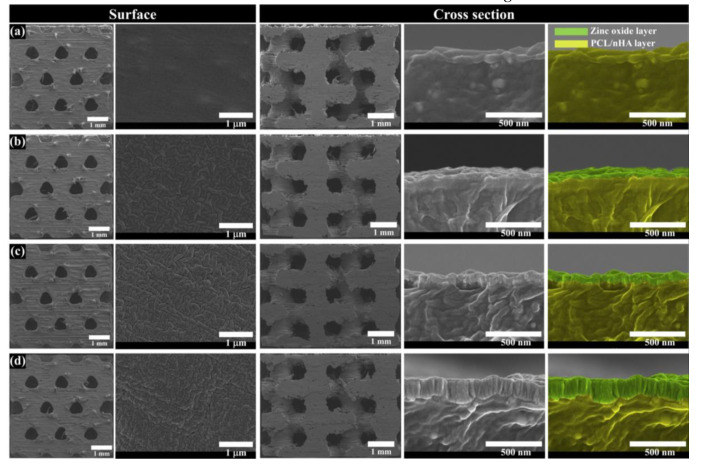
SEM images for the surface and cross-sectional views of the fabricated PCL/nHA scaffold: (**a**) PCL/nHA scaffold; (**b**) PCL/nHA-ZnO10 scaffold; (**c**) PCL/nHA-ZnO100 scaffold; (**d**) PCL/nHA-ZnO200 scaffold (green color: zinc oxide layer; yellow color: PCL/nHA layer).

**Figure 3:**
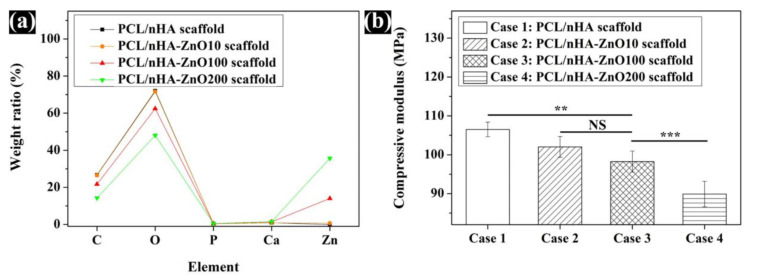
Comparison of the chemical components and mechanical property of fabricated scaffolds with varying thicknesses of zinc oxide coating: (**a**) EDS datum; (**b**) compressive modulus (NS: nonsignificant; ** *p* < 0.01; *** *p* < 0.001).

To evaluate the morphological and chemical changes of PCL/nHA scaffold by zinc oxide coating thickness, the top view, cross-sectional view, and chemical components of fabricated scaffolds were analyzed (Figure 2 and Figure 3a). In the fabricated scaffolds, uniform pore sizes and interconnectivity between pores were observed. Moreover, various thicknesses of the zinc oxide coating (10, 100, and 200 nm) were observed in the scaffolds (Figure 2b–d). The actual thickness of the zinc oxide coatings were similar to the designed thickness of the zinc oxide coatings. The chemical components of the different fabricated scaffolds were also compared (Figure 3a). In the case of the PCL/nHA scaffold, C, O, Ca, and P peaks were detected (Figure 3a). In contrast, C, O, Ca, P, and Zn peaks were detected for the PCL/nHA-ZnO10, PCL/nHA-ZnO100, and PCL/nHA-ZnO200 scaffolds. Furthermore, the Zn peak increased as the thickness of the zinc oxide layer increased (Figure 3a). As a measure of the mechanical property of the fabricated scaffolds, the compressive modulus was measured as 106.5 ± 1.8 MPa, 102.0 ± 2.6 MPa, 98.2 ± 2.7 MPa, and 89.9 ± 3.2 MPa (Figure 3b). The compressive modulus of fabricated scaffolds decreased as the thickness of the zinc oxide coating increased.

### 3.2. Evaluation of the Scaffold’s Molecular Weight, Water Absorption, and Concentration of Zinc Ions Released

**Figure 4:**
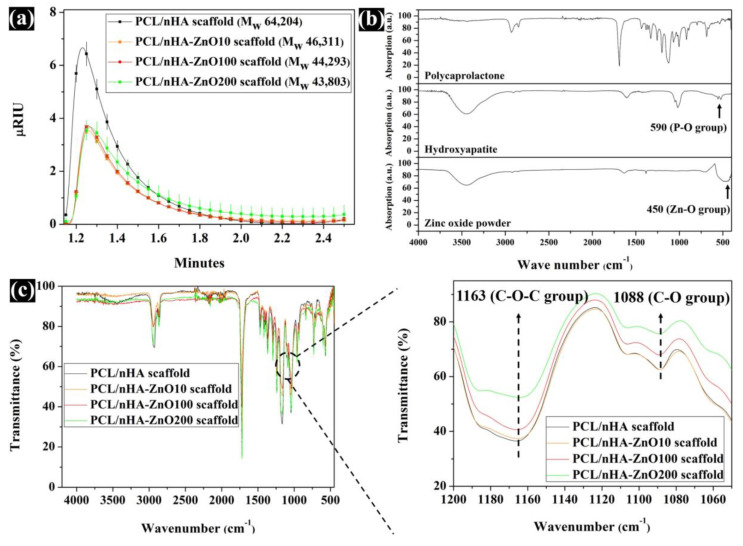
Comparison of the molecular weight and chemical composition of fabricated scaffolds: (**a**) gel permeation chromatography (GPC) results; (**b**) FT-IR results for pure material; (**c**) FT-IR results for the fabricated scaffolds.

**Figure 5:**
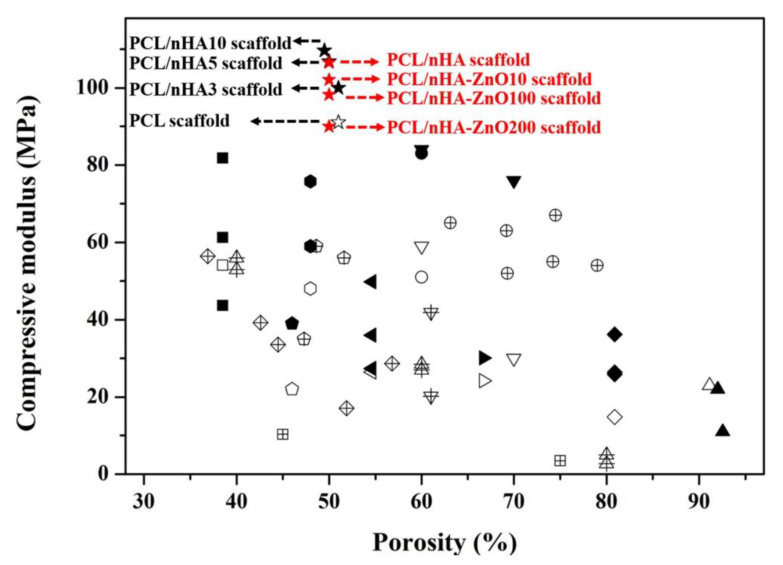
Comparison of the compressive modulus with respect to porosity of reported scaffolds (black symbols) vs proposed composite scaffold (red symbols) (the reported scaffold’s characteristics were indicated in Supplementary Table S1).

Using the sputtering system to add the zinc oxide coating decreased the compressive modulus of the PCL/nHA scaffold. To explain this phenomenon, the GPC data for molecular weight (M_w_) was measured. The measured M_w_ were 64,204, 46,311, 44,293, and 43,803. The molecular weight of the fabricated scaffolds with the added zinc oxide layer was decreased. Moreover, the µRIU values of the PCL/nHA-ZnO10, PCL/nHA-ZnO100, and PCL/nHA-ZnO200 scaffolds were lower than that of the PCL/nHA scaffold (Figure 4a). To understand the decrease in the scaffold’s molecular weight, the chemical composition of the fabricated scaffold was investigated via FT-IR (Figure 4b,c). For the chemical composition, the C–O group bends in the PCL/nHA-ZnO200 scaffold were relatively decreased compared with the PCL/nHA scaffold. The C–O (1088) and C–O–C (1163) groups in the fabricated scaffold gradually decreased as the thickness of the zinc oxide coating increased. Additionally, the compressive modulus of reported PCL or PCL/HA scaffolds with respect to porosity were investigated (Figure 5 and Supplementary Table S1). Although the compressive modulus of the proposed scaffold was decreased by adding the zinc oxide layer, the compressive modulus with respect to porosity was higher than those of reported scaffolds because of the well-dispersed composite material and kagome structure.

**Figure 6:**
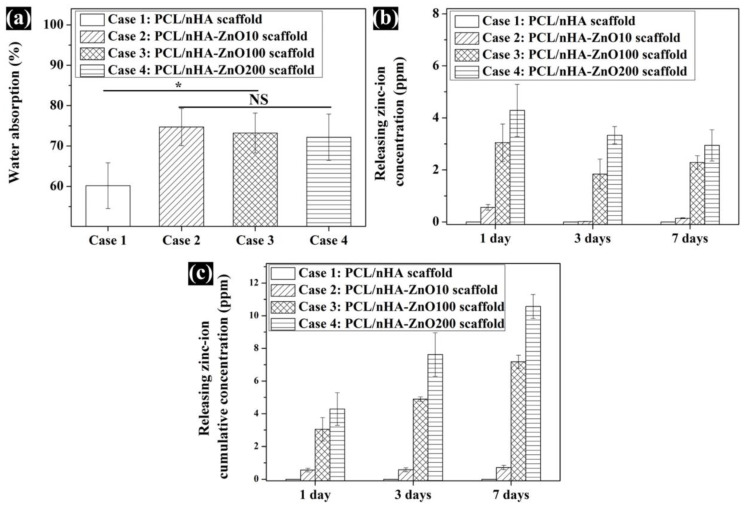
Comparison of cell response and antibacterial activity characteristics of the scaffolds: (**a**) water absorption; (**b**) concentration of zinc ions released from the fabricated scaffolds over 1 week; (**c**) cumulative concentration of zinc ions released from the fabricated scaffolds over 1 week (NS: nonsignificant; * *p* < 0.05).

To investigate the in vitro cell response and antibacterial activity characteristics of the fabricated scaffolds, the water absorption, and concentration of zinc ions released were compared (Figure 6). In regard to the in vitro cell response, the water absorption was measured as 60.1% ± 6.6%, 74.7% ± 4.6%, 73.2% ± 4.9%, and 72.1% ± 5.7% (Figure 6a). In regard to the antibacterial activity, the concentration of zinc ions released from the fabricated scaffolds were measured (Figure 6b,c). On day 1, the released zinc ion concentrations were 0, 0.5 ± 0.1, 3.0 ± 0.7 and 4.2 ± 1.0 ppm. The cumulative concentrations of zinc ions released from the fabricated scaffolds on day 7 were 0, 0.7 ± 0.1, 7.1 ± 0.4 and 10.5 ± 0.73 ppm. The concentration of zinc ions released from the fabricated scaffolds increased as the thickness of zinc oxide layer coating increased.

### 3.3. Evaluation of the Antibacterial Activity of Zinc Oxide Coating on the Fabricated Scaffolds

**Figure 7:**
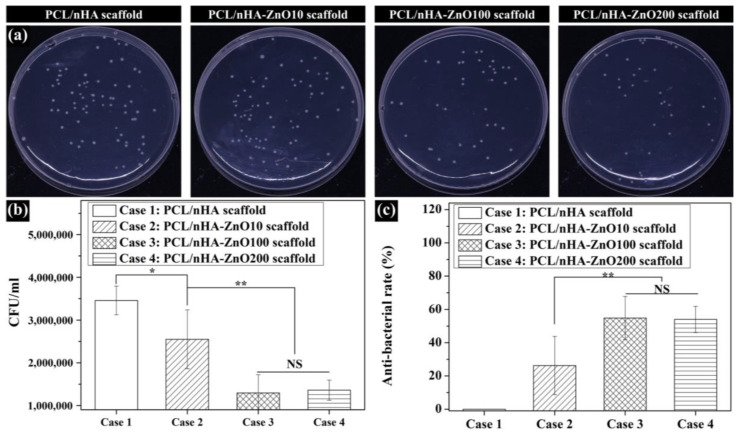
Assessment of antibacterial activity of fabricated scaffolds: (**a**) *E. coli* images recultured on agar for 24 h; (**b**) CFU; (**c**) antibacterial rate (NS: nonsignificant; * *p* < 0.05, ** *p* < 0.01).

**Figure 8:**
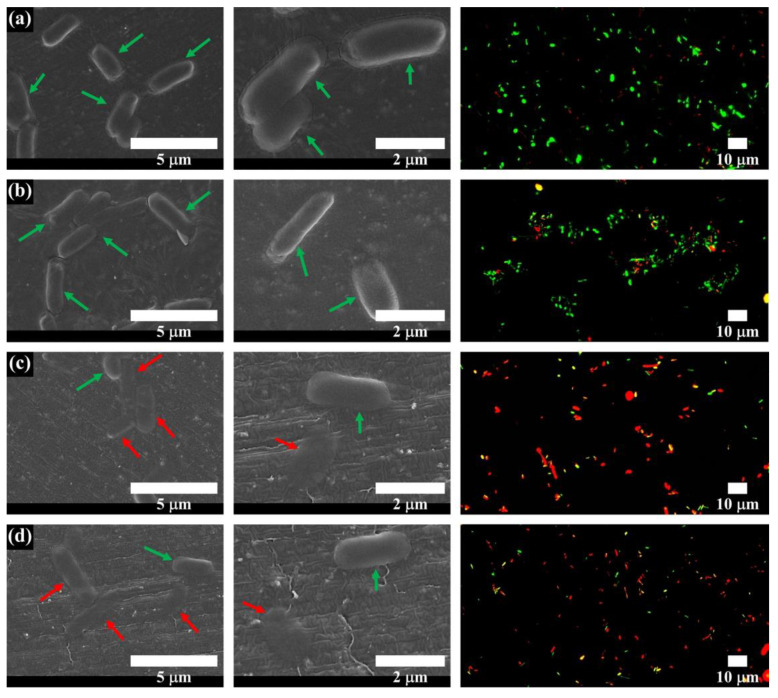
Viability of bacteria on day 1 via FE-SEM and live/dead stain: (**a**) PCL/nHA scaffold; (**b**) PCL/nHA-ZnO10 scaffold; (**c**) PCL/nHA-ZnO100 scaffold; (**d**) PCL/nHA-ZnO200 scaffold (Green arrow: live bacteria, red arrow: dead bacteria (FE-SEM); green color: live bacteria, orange color: damaged bacteria, red color: dead bacteria (live/dead)).

To assess the antibacterial activity of the fabricated scaffold according to zinc oxide coating thickness, the colony-forming units (CFUs) of *E. coli* bacteria was analyzed. The bacteria number on the PCL/nHA scaffold was similar to that on the PCL/nHA-ZnO10 scaffold (Figure 7a,b). Moreover, the bacteria numbers on the PCL/nHA-ZnO100 and PCL/nHA-ZnO200 scaffolds were lower than those of the PCL/nHA and PCL/nHA-ZnO10 scaffolds. To further identify the antibacterial activity of the fabricated scaffolds, the antibacterial rate was calculated (Figure 7c). The antibacterial rates of the PCL/nHA-ZnO10, PCL/nHA-ZnO100, and PCL/nHA-ZnO200 scaffolds are 26.2% ± 17.5%, 54.8% ± 12.9%, and 54.0% ± 7.9%, respectively. In addition, there were more dead bacteria attached to the PCL/nHA-ZnO100 and PCL/nHA-ZnO200 scaffolds than to the PCL/nHA and PCL/nHA-ZnO10 scaffolds (Figure 8). In can be concluded, therefore, that the scaffolds with the 100 or 200 nm zinc oxide coating showed higher antibacterial effects.

### 3.4. In Vitro Cell Viability and Proliferation on the Fabricated Scaffolds

**Figure 9:**
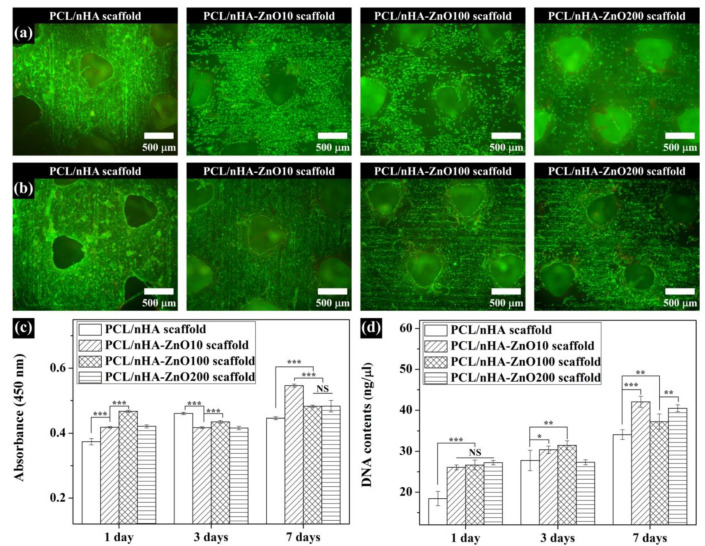
Assessment of in vitro cell response of fabricated scaffolds over 1 week: (**a**) live/dead stain images 3 days after cells were cultured on the fabricated scaffolds; (**b**) live/dead stain images 7 days after cells were cultured on the fabricated scaffolds; (**c**) CCK-8 assay; (**d**) DNA contents (NS: nonsignificant; * *p* < 0.05; ** *p* < 0.01; *** *p* < 0.001).

To investigate the effect on in vitro cell viability and proliferation according to thickness of the zinc oxide coating, we performed live/dead staining, a CCK-8 assay, and measurements of DNA content (Figure 9). For the live/dead stain, the cell viability in all scaffolds was superior after 1 week (Figure 9a,b). Furthermore, cell viability on the PCL/nHA-ZnO10, PCL/nHA-ZnO100, and PCL/nHA-ZnO200 scaffolds were higher than that of PCL/nHA scaffold on day 1 because of their zinc oxide coatings (Figure 9c,d). For the in vitro cell proliferation, the PCL/nHA-ZnO10 scaffold was superior to the other scaffolds at 1 week (Figure 9c,d). Moreover, the cell proliferation on the PCL/nHA-ZnO100 and PCL/nHA-ZnO200 scaffolds was better than that of the PCL/nHA scaffold. Therefore, cell viability and proliferation was enhanced by the zinc oxide layer coated on the fabricated PCL/nHA scaffold.

## 4. Discussion

3D-printed scaffolds with an interconnective pore size between 300–500 μm led smoothly provide nutrients and oxygen to cells [34,35,36]. Moreover, 3D-printed scaffolds with an interconnective pore size of 300–500 μm and 50% porosity enhanced the formation of essential vascularization for bone regeneration [37]. Therefore, we assessed the in vitro cell-response activities of a 3D-printed scaffold with an interconnective pore size of 500 μm, 50% porosity, and kagome structure (Table 1). In our previous study, the weight ratio of nHA in the scaffold with kagome structure was fixed as 10 wt % because the mechanical properties and in vitro cell activities of PCL/nHA scaffold (10 wt %) were superior to other scaffolds (3 wt %, 5 wt %) [31]. Moreover, this weight ratio is the maximum ratio for the printing scaffolds without fabrication problems. According to a previous study on the antibacterial activity of zinc ions, Mao et al. revealed that the zinc ion concentrations with antibacterial activity against S. aureus and *E. coli* ranged from 0.654 to 6.54 ppm on day 1 [38]. Ruml et al. reported that U-2 OS (osteosarcoma cell) viability within 2.616 ppm of the zinc ion concentration was negligible [39]. Moreover, Zhu et al. found that cell adhesion, spreading, and viability improved when the zinc ion concentration was under 5.232 ppm [40]. In this study, we investigated various thicknesses of zinc oxide coating on 3D-printed kagome-composite (PCL/nHA) scaffolds to find the adequate thickness of zinc oxide, since zinc oxide can enhance the antibacterial activity and cell response of the scaffold. We evaluated the concentrations of zinc ions released by the different scaffolds.

All the scaffolds fabricated by 3D-printing system have a uniform pore shape and pore size (Figure 2). We observed 10, 100 and 200 nm-thick zinc oxide layers on the surface of the scaffold as shown in the green-color area (Figure 2b–d). For the chemical components (Figure 3a), C, O, P, and Ca peaks were detected in the all scaffolds, however, the zinc peaks were increased as the thickness of the zinc oxide layer increased. The compressive modulus of the fabricated scaffolds decreased as the thickness of the zinc oxide layer increased (Figure 3b). To explain this phenomenon, the molecular weight of the fabricated scaffolds was measured via GPC equipment (Figure 4a). The molecular weight of the fabricated scaffolds coated with any thickness of zinc oxide was decreased compared to the PCL/nHA scaffold. To reveal the reason for this phenomenon, we investigated the chemical compositions of the fabricated scaffolds (Figure 4b,c). The detection levels of C–O (1088) and C–O–C (1163) groups in the PCL/nHA-ZnO100 and PCL/nHA-ZnO200 scaffolds were decreased compared with those in the PCL/nHA and PCL/nHA-ZnO10 scaffolds [41,42,43]. Therefore, we could assume applying the zinc oxide coating by sputtering system broke the C–O (1088) and C–O–C (1163) groups in the PCL matrix. Moreover, the molecular weight of the fabricated scaffold was decreased by the broken of C–O (1088) and C–O–C (1163) groups. According to previous studies [44,45], it was reported that the degradation rate of PCL could be accelerated by zinc oxide particles because the backbone chains of PCL were cleaved by the zinc oxide particles. Consequently, the mechanical property of the fabricated scaffold was decreased by the broken molecular chain in the PCL matrix. Although the compressive modulus of scaffolds having zinc oxide layers were decreased compared with control scaffold without zinc oxide layer, the compressive modulus of scaffold with zinc oxide layers was superior to the reported 3D-printed scaffolds because of the kagome structure and because the composite material consisted of PCL and nanosized HA (Figure 5 and Supplementary Table S1).

The fabricated scaffolds’ characteristics related to in vitro cell and antibacterial activities were investigated (Figure 6). The water absorption of the scaffold, which is related to cell adhesion and proliferation [46], was slightly increased by the presence of a zinc oxide coating. The concentration of zinc ions released from the fabricated scaffolds, which is related to the cell viability/proliferation and antibacterial activities, was investigated (Figure 6b,c). The concentration of zinc ions released increased as the thickness of the zinc oxide layer increased. We posit that the higher concentration resulted from the diffusion characteristics of the zinc oxide layer in regard to the nanoscale thickness. In terms of antibacterial activity, the PCL/nHA-ZnO100 and PCL/nHA-ZnO200 scaffolds were better than the PCL/nHA and PCL/nHA-ZnO10 scaffolds (Figure 7). In other words, the antibacterial activity occurred in the range from 3 to 4.2 ppm (the concentration of zinc ions released from the PCL/nHA-ZnO100 scaffold and the PCL/nHA-ZnO200 scaffold on day 1). This result corroborated the findings of a previously reported study [38]. Furthermore, the PCL/nHA-ZnO100 and PCL/nHA-ZnO200 scaffolds had visually more dead bacteria on them than other scaffolds (Figure 8). For the in vitro cell viability and proliferation, the cell viabilities of the PCL/nHA-ZnO10, PCL/nHA-ZnO100, and PCL/nHA-ZnO200 scaffolds were similar to that of the PCL/nHA scaffold (Figure 9). Moreover, the cell proliferations of the PCL/nHA-ZnO10, PCL/nHA-ZnO100, and PCL/nHA-ZnO200 scaffolds were higher than that of the PCL/nHA scaffold (Figure 9c,d). Although cell-proliferation of the PCL/nHA-ZnO10 scaffold was better than those of other scaffolds, the antibacterial activity of the PCL/nHA-ZnO10 scaffold was similar to the PCL/nHA scaffold. Consequently, the PCL/nHA-ZnO100 scaffold was the most appropriate for bone regeneration, considering its decreased compressive modulus, in vitro cell viability/proliferation, and antibacterial function.

## 5. Conclusions

In this study, a composite kagome scaffold with antibacterial activity fabricated by 3D-printing and a sputtering systems was proposed. Moreover, to investigate the adequate thickness of an antibacterial zinc oxide coating, we compared the characteristics, antibacterial capabilities, and cell viability/proliferation activities of fabricated scaffolds with various thicknesses of zinc oxide. Our data revealed that the compressive modulus of fabricated scaffold with a zinc oxide layer was decreased by the broken C–O–C and C–O groups in PCL molecular chain compared with the control scaffold. However, the compressive modulus of the proposed scaffold was superior to the previously reported 3D-printed scaffold because of its novel kagome structure and composite material. The antibacterial activities of the proposed scaffold with 100 or 200 nm-thick zinc oxide coating was better than those of the scaffold without zinc oxide coating or with a 10 nm-thick zinc oxide coating. Moreover, the in vitro cell viability of scaffolds with a zinc oxide layer was similar to that of the scaffold without a zinc oxide layer. Furthermore, the cell proliferation of scaffolds with a zinc oxide layer was higher than that of the scaffold without a zinc oxide layer. Consequently, we found that the proposed scaffold with a 100 nm-thick zinc oxide coating was most appropriate for bone regeneration and the prevention of surgical site infection, considering its mechanical properties, in vitro cell viability/proliferation, and antibacterial activity.

## Figures and Tables

**Table 1:** The parameters of fabricated PCL/nHA-ZnO scaffolds.

	PCL/nHA Scaffold	PCL/nHA-ZnO10 Scaffold	PCL/nHA-ZnO100 Scaffold	PCL/nHA-ZnO200 Scaffold
Target pore size	500 μm	500 μm	500 μm	500 μm
Target porosity	50%	50%	50%	50%
Sputtering time	-	56 s	562 s (9 min, 22 s)	1125 s (18 min, 45 s)
Thickness of ZnO layer	-	10 nm	100 nm	200 nm

## Supplementary Materials

The following are available online at https://www.mdpi.com/2073-4360/12/10/2193/s1, Table S1: Characteristics of reported PCL scaffolds and PCL/HA scaffolds (solid symbols indicate PCL/HA composite scaffold and blank symbols indicate PCL scaffold).
