# Supplementary material for: Evaluation of the Antibacterial Activity and Cell Response for 3D-Printed Polycaprolactone/Nanohydroxyapatite Scaffold with Zinc Oxide Coating
Source: Polymers (Basel). 2020 Sep 25;12(10):2193. doi: 10.3390/polym12102193 (PMC7601629; doi:10.3390/polym12102193)
Supplement: Supplementary file 1 [file polymers-12-02193-s001.pdf]

Supplementary

Table S1. Characteristics of reported PCL scaffolds and PCL/HA scaffolds (solid symbols indicate PCL/HA composite scaffold and blank symbols indicate PCL scaffold).

Symbol	Printing system	Structure	Material	Ref
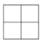	Melting extruder	Grid	PCL (M_n 80,000)	[S1]
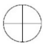	Selective laser sintering	Grid	PCL (M_w 50,000)	[S2]
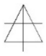	Selective laser sintering	Octagonal prism and square prism<br>Hexagonal prism and triangular prism	PCL (M_w 50,000)	[S3]
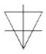	Melting extruder	Grid	PCL (M_n 80,000)	[S4]
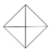	Melting extruder	Grid	PCL (M_n 45,000)	[S5]
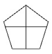	Melting extruder	Grid	PCL (M_n 70,000-90,000)	[S6]
■□	Melting extruder	Grid	PCL (M_w 50,000)<br>Nanosize HA (10-30 wt.%)	[S7]
●○	Melting extruder	Grid	PCL (M_w 43,000-50,000)<br>Microsize HA (30 wt.%)	[S8]
▲△	Melting extruder	Grid	PCL (M_w 80,000)<br>Microsize HA (40 wt.%)	[S9]
▼▽	Melting extruder	Grid	PCL (M_w 44,000)<br>Microsize HA (25 wt.%)	[S10]
◆◇	Selective laser sintering	Grid	PCL (M_n 50,000)<br>Microsize HA (10-30 wt.%)	[S11]
◀◁	Melting extruder	Grid	PCL (M_w 40,000)<br>Microsize HA (10-30 wt.%)	[S12]
▶▷	Melting extruder	Grid	PCL (M_n 65,000)<br>Nanosize HA (10 wt.%)	[S13]
⬟⬠	Melting extruder	Grid	PCL (M_w 80,000)<br>Nanosize HA (40 wt.%)	[S14]
⬢⬡	Melting extruder	Grid	PCL (M_w 50,000)<br>Nanosize HA (10-20 wt.%)	[S15]
★☆	Melting extruder<br>(Our previous study)	Kagome	PCL (M_w 43,000-50,000)<br>Nanosize HA (3-10 wt.%)	[S16]

Reference

[S1] S. Park, G. Kim, Y.C. Jeon, Y. Koh, W. Kim, 3D polycaprolactone scaffolds with controlled pore structure using a rapid prototyping system, J. Mater. Sci. Mater. Med. 20 (2009) 229–234. doi:10.1007/s10856-008-3573-4.

[S2] J.M. Williams, A. Adewunmi, R.M. Schek, C.L. Flanagan, P.H. Krebsbach, S.E. Feinberg, S.J. Hollister, S. Das, Bone tissue engineering using polycaprolactone scaffolds fabricated via selective laser sintering, Biomaterials. 26 (2005) 4817–4827. doi:10.1016/j.biomaterials.2004.11.057.

[S3] N. Sudarmadji, J.Y. Tan, K.F. Leong, C.K. Chua, Y.T. Loh, Investigation of the mechanical properties and porosity relationships in selective laser-sintered polyhedral for functionally graded scaffolds, Acta Biomater. 7 (2011) 530–537. doi:10.1016/j.actbio.2010.09.024.

[S4] D.W. Hutmacher, T. Schantz, I. Zein, K.W. Ng, S.H. Teoh, K.C. Tan, Mechanical properties and cell cultural response of polycaprolactone scaffolds designed and fabricated via fused deposition modeling, J. Biomed. Mater. Res. 55 (2001) 203–216. doi:10.1002/1097-4636(200105)55:2<203::AID-JBM1007>3.0.CO;2-7.

[S5] A.D. Olubamiji, Z. Izadifar, J.L. Si, D.M.L. Cooper, B.F. Eames, D.X.B. Chen, Modulating mechanical behaviour of 3D-printed cartilage-mimetic PCL scaffolds: Influence of molecular weight and pore geometry, Biofabrication. 8 (2016) 1–18. doi:10.1088/1758-5090/8/2/025020.

[S6] J.F.M. Ribeiro, S.M. Oliveira, J.L. Alves, A.J. Pedro, R.L. Reis, E.M. Fernandes, J.F. Mano, Structural monitoring and modeling of the mechanical deformation of three-dimensional printed poly(ϵ-caprolactone) scaffolds, Biofabrication. 9 (2017) 25015. doi:10.1088/1758-5090/aa698e.

[S7] M.H. Kim, C. Yun, E.P. Chalisserry, Y.W. Lee, H.W. Kang, S.H. Park, W.K. Jung, J. Oh, S.Y. Nam, Quantitative analysis of the role of nanohydroxyapatite (nHA) on 3D-printed PCL/nHA composite scaffolds, Mater. Lett. 220 (2018) 112–115. doi:10.1016/j.matlet.2018.03.025.

[S8] E. Nyberg, A. Rindone, A. Dorafshar, W.L. Grayson, Comparison of 3D-Printed Poly-ε-Caprolactone Scaffolds Functionalized with Tricalcium Phosphate, Hydroxyapatite, Bio-Oss, or Decellularized Bone Matrix, Tissue Eng. Part A. 23 (2016) 503–514. doi:10.1089/ten.tea.2016.0418.

[S9] S.A. Park, S.H. Lee, W.D. Kim, Fabrication of porous polycaprolactone/hydroxyapatite (PCL/HA) blend scaffolds using a 3D plotting system for bone tissue engineering, Bioprocess Biosyst. Eng. 34 (2011) 505–513. doi:10.1007/s00449-010-0499-2.

[S10] L. Shor, S. Güçeri, X. Wen, M. Gandhi, W. Sun, Fabrication of three-dimensional polycaprolactone/hydroxyapatite tissue scaffolds and osteoblast-scaffold interactions in vitro, Biomaterials. 28 (2007) 5291–5297. doi:10.1016/j.biomaterials.2007.08.018.

[S11] S. Eshraghi, S. Das, Micromechanical finite-element modeling and experimental characterization of the compressive mechanical properties of polycaprolactone-hydroxyapatite composite scaffolds prepared by selective laser sintering for bone tissue engineering, Acta Biomater. 8 (2012) 3138–3143. doi:10.1016/j.actbio.2012.04.022.

[S12] K.S. W. Jiang, J. Shi, W. Li, Morphology, wettability, and Mechanical properties of polycaprolactone/hydroxyapatite composite scaffolds with interconnected pore structures fabricated by a mini-deposition system, Polym. Eng. Sci. (2011) 1–5. doi:10.1002/pen.

[S13] J.Y. Kim, T.J. Lee, D.W. Cho, B.S. Kim, Solid free-form fabrication-based PCL/HA scaffolds fabricated with a multi-head deposition system for bone tissue engineering, J. Biomater. Sci. Polym. Ed. 21 (2010) 951–962. doi:10.1163/156856209X458380.

[S14] B. Dorj, J.E. Won, J.H. Kim, S.J. Choi, U.S. Shin, H.W. Kim, Robocasting nanocomposite scaffolds of poly(caprolactone)/hydroxyapatite incorporating modified carbon nanotubes for hard tissue reconstruction, J. Biomed. Mater. Res. - Part A. 101 A (2013) 1670–1681. doi:10.1002/jbm.a.34470.

[S15] B. Huang, G. Caetano, C. Vyas, J.J. Blaker, C. Diver, P. Bártolo, Polymer-ceramic composite scaffolds: The effect of hydroxyapatite and β-tri-calcium phosphate, Materials (Basel). 11 (2018). doi:10.3390/ma11010129.

[S16] Y.S. Cho, M. Quan, N.U. Kang, H.J. Jeong, M.W. Hong, Y.Y. Kim, Y.-S. Cho, Strategy for enhancing mechanical properties and bone regeneration of 3D polycaprolactone kagome scaffold: Nano hydroxyapatite composite and its exposure, Euro. Polym. 134 (2020). doi.org/10.1016/j.eurpolymj.2020.109814.
